# Generalized Einstein Equation for Ceramics Suspension Rheology

**DOI:** 10.1021/acsomega.6c04319

**Published:** 2026-07-13

**Authors:** Petr Ptáček, František Šoukal, Tomáš Opravil, Tina Skalar, Jan Blahut, David Markusík, Patrik Sokola

**Affiliations:** † Vysoke Uceni Technicke v Brne Fakulta Chemicka, Brno 61200, South Moravian Region, CZ; ‡ University of Ljubljana, Faculty of Chemistry and Chemical Technology, Ljubljana 1000, Slovenia; § Akademie Ved Ceske Republiky, Brno 61200, South Moravian Region, CZ

## Abstract

This work introduces the Generalized Einstein-type Equation Rheological Gaussian Model (E^2^RG) for suspension rheology, extending the linear Einstein-type form to concentrated systems while preserving the correct Einstein limit at very low volume fractions. The formulation enables accurate prediction of relative viscosity across the entire packing interval from φ = 0 to φ_max_. Unlike empirical power-law or exponential approaches, E^2^RG does not apply an exponent to particle concentration; instead, it modulates the entire Einstein expression through a correction based on the Gaussian error function, providing a smooth, physically consistent transition from ideal to nonideal regimes without introducing arbitrary constants. A key advantage of E^2^RG is its intrinsic verifiability: the model parameters retain clear physical meaning, reflecting the intensity of particle interactions rather than serving as free-fitting constants. After applying the correction term, linearization enables verification of whether the fit has correctly separated the ideal contribution from interaction effects, providing an internal self-consistency check of the model decomposition, confirming that the two parameters fulfill their intended roles rather than acting as compensating free variables. Beyond its mathematical robustness, the E^2^RG formulation is consistent with the Central Limit Theorem (CLT), since the Gaussian correction is consistent with the Gaussian limit expected from the cumulative effect of multiple weak constraints of many weak, multiplicative interaction constraints acting at the particle scale. The transition at φ_max._ aligns with the jamming framework, offering a physically coherent interpretation of flow cessation without invoking a true viscosity divergence. Together, these features distinguish E^2^RG from conventional empirical models and make it a conceptually transparent and practically versatile tool for describing suspension flow behavior.

## Graphical abstract

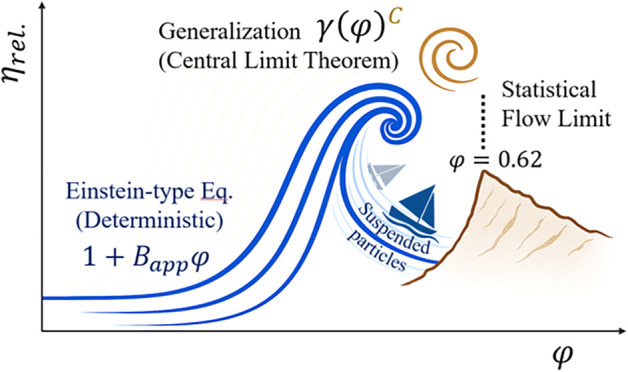

## Introduction

Suspension rheology plays a critical role in numerous industrial and scientific applications, including ceramic processing,^1,2^ coatings,^3^ and advanced manufacturing.^4,5^ Accurate prediction of viscosity as a function of particle concentration is essential for controlling flow behavior and ensuring process reliability.^6^ This requirement has become even more critical with the advent of additive manufacturing and 3D printing of ceramics and composites, where precise rheological control determines layer uniformity, dimensional accuracy, and defect-free consolidation.^7–9^ In these processes, the ability to predict and adjust viscosity across a wide concentration range directly influences printability, structural integrity, and final material performance.

Classical approaches to modeling suspension viscosity date back to Einstein’s pioneering work, which introduced a linear relationship between relative viscosity and particle volume fraction for dilute systems. This formulation, expressed as^10–12^

1 $$\eta_{\mathrm{rel}.}^{E}=1+B\varphi$$

where η_rel._^*E*^ is the relative (Einstein) viscosity of the diluted suspension, and *B* is the intrinsic viscosity coefficient. Under ideal conditions, specifically for noninteracting spherical particles, the value of *B* is equal to 2.5.^13,14^ Einstein’s equation is applicable for volume fractions (φ) below 0.02, where particle interactions can be considered negligible.^15,16^

Over the past decades, numerous extensions of Einstein’s equation have been proposed to describe concentrated suspensions. As particle concentration increases beyond the infinitely dilute regime, hydrodynamic interactions between pairs of particles become important. Batchelor and Green^17^ extended Einstein’s theory by including a term of order φ^2^, which accounts for pairwise interactions in dilute to moderately concentrated suspensions and improves agreement with experiments up to φ ≲ 0.10.^18,19^ Mooney,^20^ using an exponential approach, incorporates crowding and packing effects by recognizing that each incremental addition of particles occurs in a continuously changing medium.^21^ Finally, Krieger–Dougherty^22^ presented a semiempirical extension based on the power law, which diverges at a maximum packing fraction.^23^ As the need to describe the complexity of shape, size distribution, and interaction effects in complex ceramic suspensions has increased, modified Krieger–Dougherty forms have been developed.^24,25^ While these approaches improve predictive accuracy, they often rely on empirical adjustments or assume unrealistic conditions, such as infinite viscosity at the packing limit.^26,27^ Such assumptions do not fully capture the physical nature of flow constraints arising from complex particle interactions and geometric limitations.

To address these challenges, this work introduces the Generalized Einstein Equation (E^2^RG), a model that extends Einstein’s formulation across the entire packing interval without resorting to arbitrary constants or empirical divergence. The E^2^RG model incorporates a Gaussian weighting factor that captures the cumulative effect of particle-interaction constraints, enabling a smooth and physically well-behaved transition from ideal to nonideal regimes. Unlike traditional models, E^2^RG maintains conceptual clarity by preserving the Einstein limit while embedding interaction-driven complexity in a mathematically consistent manner.

## Experimental Procedure

8Y-ZrO_2_ powder (ZirPro, France) with *d*_50_ = 300 nm was selected as a representative industrially used material. The X-ray diffraction (XRD) analysis (Figure 1) is included to confirm the phase composition and material consistency of the studied system prior to rheological evaluation. Measurement was performed on an Empyrean diffractometer (Malvern Panalytical, U.K.). The measurement was performed using Cu Kα_1_ radiation (λ = 1.540598 Å). As a dispersion medium, polyethylene glycol PEG with *M*_w_ = 400 000 (Merck KGaA, Germany), was used. To ensure the homogeneity of the suspension, 7 wt % of surfactant Disperbyk −103 (BYK Chemie, Germany), respecting the weight of the powder, was used. Suspensions were prepared as follows:

- A mass of 30 ± 1 g of zirconium milling balls (1 cm diameter) was weighed into the polypropylene cup (57 mm inner diameter; 45 mm depth).
- 25 g of dispersion medium, polyethylene glycol, was added to the cup.
- 7 wt % of dispersant Disperbyk-103, respecting the weight of the powder, was added to the cup.
- One-third of an 8Y-ZrO_2_ powder was added, and the mixture was mixed at 1200 rpm for 10 min in a planetary mixer, Thinky ARE 320 (Donau Lab, Czech Republic).
- Immediately after homogenization, a defoaming regime (2400 rpm for 30 s) followed.
- Two previous steps were repeated twice until the required amount of powder was reached.
- A set of suspensions with volume fractions φ = 0, 2.5, 5, 7.5, 10, 15, 20, 25, 30, 35, and 40 vol % was prepared.

**Figure 1:**
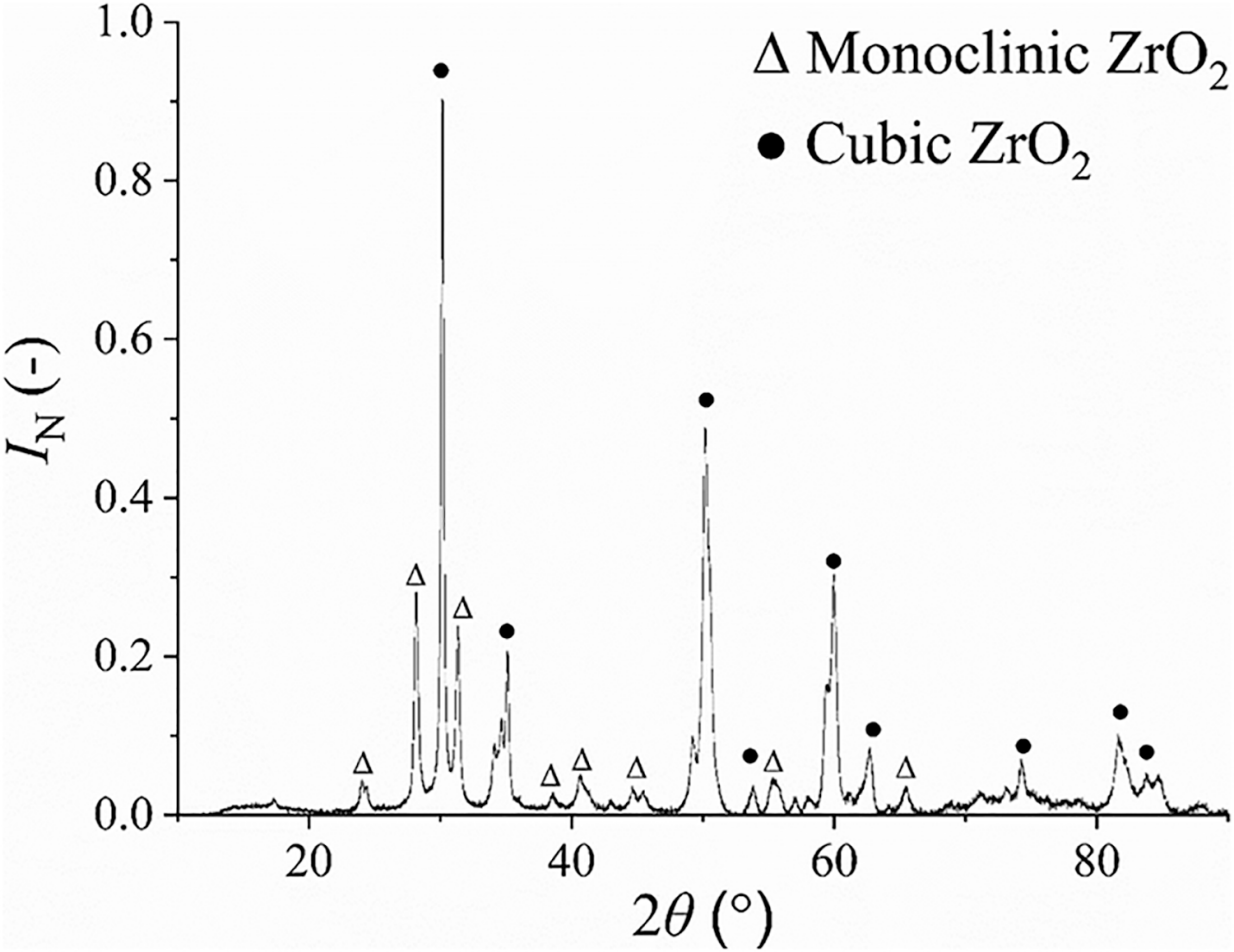
XRD spectrum of 8Y-ZrO_2_ powder.

All rheological measurements were carried out using a Discovery HR-2 rheometer (TA Instruments, USA) equipped with a stainless-steel concentric-cylinder geometry. The sample was tempered for 6 min at the set temperature using a Peltier lower plate, followed by a preshear step. Subsequently, the tempered sample underwent a 60 s flow peak-hold step, with data recorded at 5 s intervals. All measurements were conducted at temperatures of 25, 30, 35, 40, and 50 °C under a constant shear rate of 50 s^–1^.

## Results and Discussion

### Derivation of the Fitting Equation

This section presents the step-by-step derivation of the E^2^RG (Einstein Equation–Generalized) formulation, starting from the classical Einstein-type equation and introducing the Gaussian correction that enables accurate fitting across the full concentration range. E^2^RG abbreviation highlights two key aspects:

(1) The model originates from the classical Einstein equation for suspension rheology,
(2) It incorporates a Gaussian correction based on Gaussian statistics, extending its applicability across the full range of volume fractions.

The term E^2^RG offers a concise and practical way to refer to the formulation, while preserving clarity regarding its conceptual foundation.

The E^2^RG formulation is rooted in the classical Einstein equation for dilute suspensions (eq 1). Its limiting form (eq 2) later became known as the Einstein limit, a term introduced in subsequent rheological literature^28–30^ to denote the limit behavior of dilute suspensions

2 $$\lim_{\varphi \to 0}\left(\eta_{\mathrm{rel}.}\right)=1$$

and is respected by all known rheological models for suspensions, even at volume fractions beyond the original validity range of Einstein’s eq (eq 1). To extend this relationship across the full range of volume fractions, a Gaussian correction is introduced via the Gaussian error function,^31^ which serves as a Gaussian weighting factor that modulates the Einstein relation according to the statistical intensity of particle-interaction constraints

3 $$\gamma =-\frac{1}{\mathrm{erf}\left(\ln\left(\varphi \right)\right)}$$

eq 3 represents the least-deforming and maximally smooth realization of a Gaussian correction, since the error function provides a particularly smooth and mathematically convenient realization among infinitely differentiable sigmoidal functions. Sigmoid-curve without singularities that simultaneously satisfies the natural boundary conditions, γ→1 as φ→0 and, γ→*∞* as φ→1*^–^*. In E^2^RG, γ­(φ) therefore acts as the smoothest statistically emergent transition from the Einstein limit toward the hypothetical φ→1 state, while the physical saturation of the suspension occurs earlier at φ_max._ ≈ 0.62. The use of the error function should therefore be understood as a statistically motivated and physically consistent modeling choice, rather than as a uniquely derived consequence of a specific probabilistic formulation. It should be emphasized that the present formulation operates at the level of relative viscosity as a macroscopic transport property. In this representation, detailed micromechanical interactions are implicitly incorporated into the effective response of the system rather than explicitly resolved.

The Gaussian interaction term assumes that each particle in a concentrated suspension is subjected to many weak, statistically independent microconstraints arising from crowding, DLVO forces, hydrodynamic shielding, and geometric hindrance.^32–34^ Because these constraints accumulate multiplicatively,^35^ their logarithmic combination can be interpreted as approaching a Gaussian-type form under Central Limit Theorem-type arguments, making ln φ the natural coordinate of the correction. The resulting cumulative weighting function *erf*(ln *φ*) provides a smooth and physically consistent deformation of the Einstein-type baseline across the entire concentration range. With ln φ < 0 for all φ ∈ (0,1), the leading minus sign ensures γ­(φ) > 1, preserving the correct orientation of the correction. In summary, eq 3 represents a statistically grounded Gaussian weighting factor emerging from the accumulation of many small interaction constraints.

The choice of the Gaussian error function (erf) as the weighting term in eq ([Disp-formula eq3]) can be compared with other smooth sigmoidal functions, such as the logistic function and hyperbolic tangent, which may also provide smooth transitions between dilute and concentrated regimes. In the present formulation, erf is selected because it consistently satisfies the key boundary conditions imposed by the model: (i) γ­(φ) → 1 as φ → 0 (Einstein limit), (ii) γ­(φ) → ∞ as φ → 1^–^ (formal asymptotic divergence), and (iii) smooth, infinitely differentiable behavior across the full range.

From a statistical perspective, this choice is further supported by its relation to Gaussian cumulative behavior, providing a natural representation of the cumulative effect of multiple interaction constraints in the transformed (logarithmic) variable. This selection is not uniquely determined by the underlying physics. Rather, the error function represents a simple and physically consistent choice within a broader class of admissible sigmoidal functions.

The function γ­(φ) acts as a Gaussian weighting factor that multiplicatively modifies the ideal Einstein relation, reflecting the cumulative statistical influence of particle-interaction constraints. At the Einstein limit defined by eq ([Disp-formula eq2]), γ(0)→1

4 $$\lim_{\varphi \to 0}\left(\gamma \right)=1=\lim_{\varphi \to 0}\left(\eta_{\mathrm{rel}.}\right)$$

In this dilute regime, the Gaussian weighting factor approaches unity smoothly, and all of its derivatives vanish,^36^ ensuring that the Gaussian correction preserves both the Einstein limit and its local linear structure.

This means that a pure solvent (no dispersed phase) and a suspension with zero concentration exhibit the same rheological behavior with respect to particle contribution: the dispersed phase plays no role in momentum transfer because there are no particles to interact.^37^ The solvent transmits momentum normally, and the correction factor remains neutral, ensuring full consistency with the Einstein limit. As φ→1^–^, the Gaussian interaction term, and, consistently with its structure, all of its derivatives, diverge

5 $$\lim_{\varphi \to 1^{-}}\left(\gamma \right)=\infty$$

This divergence represents a purely mathematical limit, distinct from the Statistical Flow Limit (eq 9). While φ_max_ marks the onset of macroscopic flow cessation, the formal limit φ→1^–^ corresponds to idealized geometric closure at which even microscopic rearrangements would cease.

The probability of successful particle-mediated momentum transfer becomes infinitesimally small as the Gaussian correction factor tends to infinity (eq 5).^38^ This divergence does not imply that the viscosity of a pure solid becomes infinite; rather, it reflects a statistical condition where the dispersed phase ceases to contribute to flow. Viscosity cannot be meaningfully defined as a transport property of a rigid solid, making this interpretation a physically consistent alternative to the classical concept of “infinite viscosity”, which does not consider the change of flow mechanism beyond the packing threshold.

Building on eq 4, the combination of the Gaussian weighting factor (eq 3) with the Einstein eq (eq 1) yields the generalized formulation of E^2^RG

6 $$\eta_{\mathrm{rel}.}=\eta_{\mathrm{rel}.}^{\mathrm{EF}}\cdot \gamma^{C}=\left(1+B_{\mathrm{app}.}\varphi \right){\left(-\frac{1}{\mathrm{erf}\left(\ln\varphi \right)}\right)}^{C}$$

Here, the parameter *B*_app._ represents the apparent intrinsic viscosity, i.e., the effective linear slope of the Einstein-type baseline after removing the Gaussian weighting. It reflects the deterministic (concentration-controlled) contribution of particle interactions. In this sense, *B*_app._ is not directly comparable to the intrinsic viscosity coefficient *B* in the classical Einstein relation, as it is defined within a decomposed formulation that incorporates interaction effects through the Gaussian weighting factor.

The scaling exponent *C* denotes the scaling exponent of the Gaussian deformation, quantifying the strength of the cumulative, statistically emergent constraints expressed by the Gaussian factor γ­(φ). Together, *B*_app_. and *C* form the deterministic and Gaussian components of the E^2^RG model, respectively. Upper index EF denotes the Einstein Form, defined as

7 $$\eta_{\mathrm{rel}.}^{\mathrm{EF}}=1+B_{\mathrm{app}}\varphi$$

This notation emphasizes that the structure of the dilute baseline remains Einstein-like, while the slope represents the system-specific, collective behavior of correlated particle groups rather than the ideal single-particle intrinsic viscosity *B* = 2.5. The Einstein Form therefore preserves the conceptual role of the linear baseline without implying that *B*_app_ is a modified version of *B*, but instead reflects the effective contribution of interacting particle assemblies characteristic for real suspensions.

Figure 2 illustrates how the Gaussian weighting factor γ­(φ) scales the Einstein-type baseline (eq 7) across the full concentration range: γ→1 at the Einstein limit (eq 4), rises to γ­(φ_max._) = 2 at the Statistical Flow Limit φ_max_ ≈ 0.62, and formally diverges as φ→1^–^(eq 5). The schematic comparison between *B* and *B*_app._ emphasizes that *B*_app._ represents the collective contribution of correlated particle groups rather than the ideal single-particle value *B*, reinforcing that eq 7 captures system-specific interaction effects. The screw metaphor serves as a stylized secondary *y*-axis: its increasing turns represent the monotonic growth of γ­(φ), and its vertical elevation reflects the cumulative scaling relation (eq 6), with the exponent *C* transforming the Gaussian rise of γ­(φ) into the accelerated increase characteristic of concentrated suspensions.

**Figure 2:**
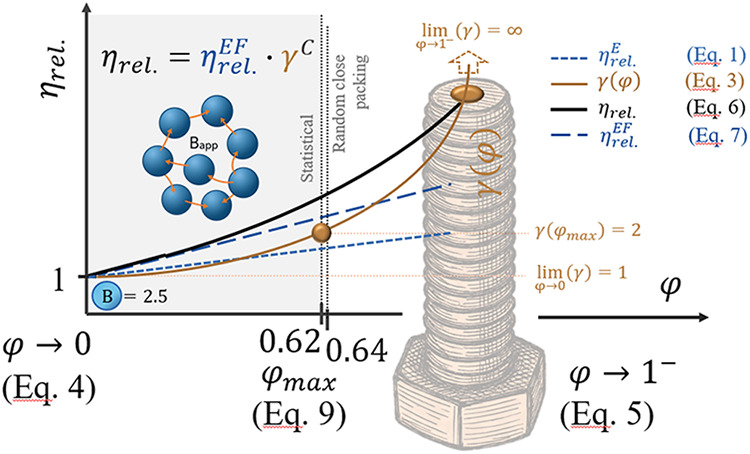
Schematic illustration of the relative viscosity η_rel._(φ) is shown as the allometrically scaled result of the deterministic Einstein-type baseline and the statistical interaction factor γ­(φ)^*C*^.

Furthermore, this work introduces the term Gaussian Allometric Evolution Law (GAEL) as a descriptive expression of the multiplicative structure arising within the present formulation. In this context, a deterministic initial condition defines the concentration-controlled baseline (1|*B*_app._φ), while a Gaussian weighting factor γ­(φ)^*C*^ captures the subsequent modification of the system response under interaction-driven constraints

$$\mathrm{GAEL}=d\mathrm{eterministic}\;i\mathrm{nitial}\;c\mathrm{ondition}\times e\mathrm{volution}\;{l\mathrm{aw}}^{\mathrm{scaling}\;\mathrm{exponent}}$$

The GAEL structure adopted here reflects a general statistical tendency: when numerous weak and heterogeneous microprocesses interact multiplicatively, their cumulative effect can be described by a smooth Gaussian-like contribution in the transformed variable, in a manner consistent with CLT-based considerations.^38–40^ When embedded into the deterministic Einstein-type baseline, this Gaussian component leads to a nonlinear increase in viscosity with concentration, consistent with the observed behavior of interacting suspensions. In this sense, GAEL does not represent a general theoretical principle, but serves as a convenient description of the interplay between deterministic and nonlinear contributions in the present model.

eq 6 (η_rel._ = η_rel._^*EF*^γ^*C*^) may be viewed as the integrated solution of a simple first-order separable differential relation. Taking logarithms yields ln η_rel._ = ln η_rel._^*EF*^ + *C* ln γ­(φ), which directly implies

8 $$\frac{d\eta_{\mathrm{rel}.}}{\eta_{\mathrm{rel}.}}=C\cdot \frac{d\gamma }{\gamma }$$

This differential form expresses a central principle of the GAEL framework: changes in relative viscosity are intrinsically relative, not absolute; they scale in proportion to the current state of the system with respect to the Gaussian statistic constraint. In discrete terms, $\frac{\Delta \eta_{\mathrm{rel}.}}{\Delta \gamma }\approx C\cdot \frac{\eta_{\mathrm{rel}.}}{\gamma }$ , showing that each incremental deformation depends on the ratio of the current viscosity to the prevailing Gaussian constraint. The system thus evolves through state-dependent, multiplicatively coupled increments rather than through an additive superposition rule. In the presented sense, eq 6 embodies an allometric growth law in which deterministic and statistical contributions remain dynamically coupled throughout the evolution of the suspension.

### Statistical Flow Limit

The Statistical Flow Limit (SFL) is defined as the concentration at which the Gaussian weighting factor γ­(φ), introduced in eq 3, is defined as the concentration at which the, i.e.

9 erf(ln⁡φ)=−0.5⇒φ≈0.62

Within the present formulation, this condition represents a model-defined transition point at which the contributions captured by the statistical weighting become balanced, leading to the loss of coherent macroscopic transport. Importantly, this value is not introduced as an independent physical constant, but follows directly from the functional structure of eq 3. Its role is therefore interpretive within the model, rather than predictive in a universal sense. While the numerical proximity of φ ≈ 0.62 to values reported for random close packing is noted,^41,42^ it is not imposed as a constraint in the formulation. In addition, real suspension systems may exhibit system-dependent variations of packing-related limits. In the present model, such variability is reflected through the system-specific parameters (*B*_app._ and *C*), which govern how the system approaches this statistical transition. The SFL should therefore be understood as a consistent reference point within the model structure, rather than as a universal packing limit. This regime is not directly covered by the experimental data presented here and should thus be interpreted as a model-based extrapolation.

At this transition point, the model indicates a loss of coherent macroscopic transport, although microscopic rearrangements may still occur. In this sense, the Statistical Flow Limit represents a model-defined transition separating regimes of effective transport from transport-limited behavior. Importantly, the formal divergence of γ­(φ) as φ → 1^–^ (eq 5) reflects a mathematical property of the Gaussian weighting function rather than a physically realizable flow condition. The physically relevant transition therefore occurs at the Statistical Flow Limit, well before the asymptotic divergence is approached. This distinction separates the E^2^RG formulation from classical models, in which divergence is directly associated with the flow limit. In E^2^RG, the divergence remains part of the mathematical structure, but does not define the onset of flow cessation.

Classical singular models (Krieger–Dougherty, Quemada) place a divergence at φ_max._, but this divergence can be interpreted as an asymptotic approximation of the steep rise toward the SFL, not as a physically attainable state. A true divergence at φ_max._ would imply the input of mechanical work without any dissipative pathway, contradicting the fact that dense suspensions always retain microscopic dissipation through intermittent granular rearrangements and microslip.^43,44^ The SFL therefore provides a physically consistent alternative: macroscopic flow ceases because it is statistically impossible, while microscopic mobility remains possible. This aligns naturally with the concept of marginal jamming, where a constrained contact network supports stress but cannot reorganize into a coherent global flow.^45–47^

### Comparative Analysis with Einstein-Based Extensions

This section contrasts the E^2^RG formulation with classical extensions of Einstein’s equation, focusing on their conceptual foundations. Traditional models such as Batchelor’s polynomial correction^17^

10 $$\eta_{\mathrm{rel}.}=1+2.5\;\varphi +6.2\;\varphi^{2}$$

and the Krieger–Dougherty power-law formulation^22^

11 $$\eta_{rel.}={\left(1-\frac{\varphi }{\varphi_{\max.}}\right)}^{-B\varphi_{\max.}}$$

represent deterministic concentration-based extensions of the Einstein relation. These classical models extend Einstein’s relation through deterministic concentration-based scaling. In contrast, E^2^RG employs a Gaussian weighting factor reflecting the statistical likelihood of momentum-transfer events. The comparison below outlines the conceptual differences between these formulations. Here, *B* denotes the Einstein intrinsic viscosity coefficient corresponding to the ideal dilute limit, and should not be confused with the parameter *B*_app._ used in the present formulation.

Since eq 11 reduces to eq 1 in the dilute limit, its Taylor expansion for φ→0

12 $$\eta_{rel.}=1+B\varphi +\frac{B\varphi^{2}\left(B\varphi_{\max}.+1\right)}{2\varphi_{\max.}}\cdot \cdot \cdot \eta_{rel.}={\left(1-\frac{\varphi }{\varphi_{\max.}}\right)}^{-B\varphi_{\max.}}$$

confirms that both eqs 10 and[Disp-formula eq11] preserve the Einstein limit (eq 2) and can be regarded as direct extensions of the original formulation. This makes them natural reference models for comparison with E^2^RG.

The E^2^RG model generalizes Einstein’s equation by preserving its dilute-limit behavior while providing a smooth extension into the concentrated regime. Classical Einstein-based extensions, such as Batchelor’s polynomial correction (eq 10) for pairwise hydrodynamic interactions^17,48^ or Mooney’s exponential formulation capturing crowding effects, retain a deterministic concentration-scaling structure^20^ and are valid only within specific concentration ranges. The Krieger–Dougherty eq (eq 11) adds a formal divergence at φ_max_ and typically relies on fixed empirical parameters, which may limit its physical interpretability.^49,50^ In contrast, E^2^RG replaces concentration-based scaling with a Gaussian weighting factor that reflects the statistical likelihood of momentum-transfer events under interaction-driven constraints, yielding a conceptually distinct and physically grounded description toward dense packing.

### Physical Consistency of the Fit

The parameters *B*_app._ and *C* represent two complementary projections of the same interaction-driven phenomenon: *B*_app._ captures the deterministic amplification of the Einstein baseline, whereas *C* expresses its stochastic counterpart through the depth of the Gaussian correction. Although both parameters originate from the same physical interaction intensity, eq 6 separates them cleanly into deterministic and statistical components. In this form, the expression becomes linear in φ, enabling direct extraction of *B*_app._ from the intercept-fixed representation

13 $$\frac{\eta_{\mathrm{rel}.}}{\gamma^{C}}=1+B_{\mathrm{app}.}\varphi$$

The intercept is fixed at unity corresponding to the Einstein limit (η_rel._ = 1 as φ→0, see eq 4). The slope of the resulting straight line directly represents the apparent Einstein coefficient *B*_app._, which quantifies the ideal contribution adjusted for interaction effects. The rearranged form $\frac{\eta_{\mathrm{rel}.}}{\gamma^{C}}-B_{\mathrm{app}.}\varphi =1$ represents an alternative expression of the Einstein limit (eq 4). This shows that linearization does not replace the original concept but confirms its deterministic foundation after removing the stochastic correction. This linearization constitutes an internal model-consistency check: it confirms that the two-parameter decomposition in eq 6 is self-consistent and that the extracted *B*_app._ retains its intended physical role as the deterministic Einstein-form slope after the stochastic Gaussian correction has been removed. It does not represent an independent experimental validation of the parameter values, which would require, for example, comparison of *B*_app._ against intrinsic viscosity measurements at infinite dilution or independent microstructural characterization of particle interaction intensity. Within the scope of the present work, the high *R* ^2^ of the linearized fit demonstrates internal consistency of the model decomposition and indicates that the parameters are not acting as compensating free variables. It is important to distinguish the role of *B*_app._ and *C* from that of independently measurable physical constants. Neither parameter is directly derivable from first-principles calculations or independently accessible through nonrheological measurements in the general case of complex ceramic suspensions. Rather, both are system-specific quantities that must be extracted by fitting eq ([Disp-formula eq6]) to experimental viscosity data spanning a sufficient concentration range. In this respect, the E^2^RG model is semiempirical: the functional form, including the choice of the error function, the form of γ­(φ), and the value of φ_max_, is fixed by the statistical framework and requires no fitting, while *B*_app._ and *C* are determined from data. The physical significance of *B*_app._ and *C* lies not in their being independently predictable, but in their interpretability: *B*_app._ quantifies the effective Einstein-type contribution of interacting particle groups, and *C* characterizes the intensity of cumulative stochastic constraints. Their mutual consistency, confirmed through the linearization procedure (eq 13), ensures that the fitted values reflect genuine physical features of the suspension rather than arbitrary numerical compensation. Researchers intending to apply the model to a new system should treat *B* ^app^. and *C* as system-specific characterization parameters.

### Experimental Verification

Figures 3 and 4 present representative plots of relative viscosity as a function of volume filling together with the corresponding verification of fit consistency. Figure 3 shows the results obtained for the 8Y-ZrO_2_ - PEG suspensions, whereas Figure 4 displays the data for silica sand (Dorsilit) dispersed in carboxymethylcellulose (CMC), using rheological data reported in Sokola et al.^51^

**Figure 3:**
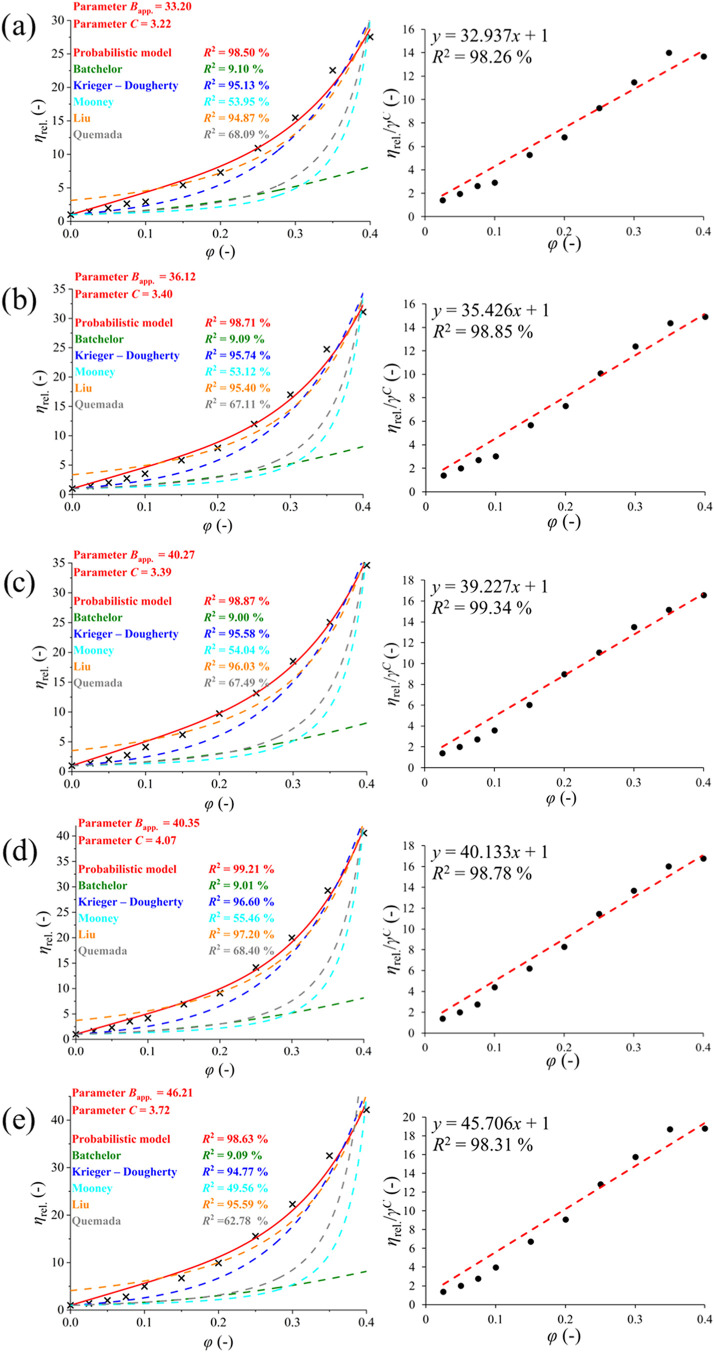
Graph of the dependence of relative viscosity on volume filling (left) with evaluation of physical consistency of the fit (right) for 8Y-ZrO_2_ −PEG suspensions for (a) 25 °C; (b) 30 °C; (c) 35 °C; (d) 40 °C; (e) 50 °C.

**Figure 4:**
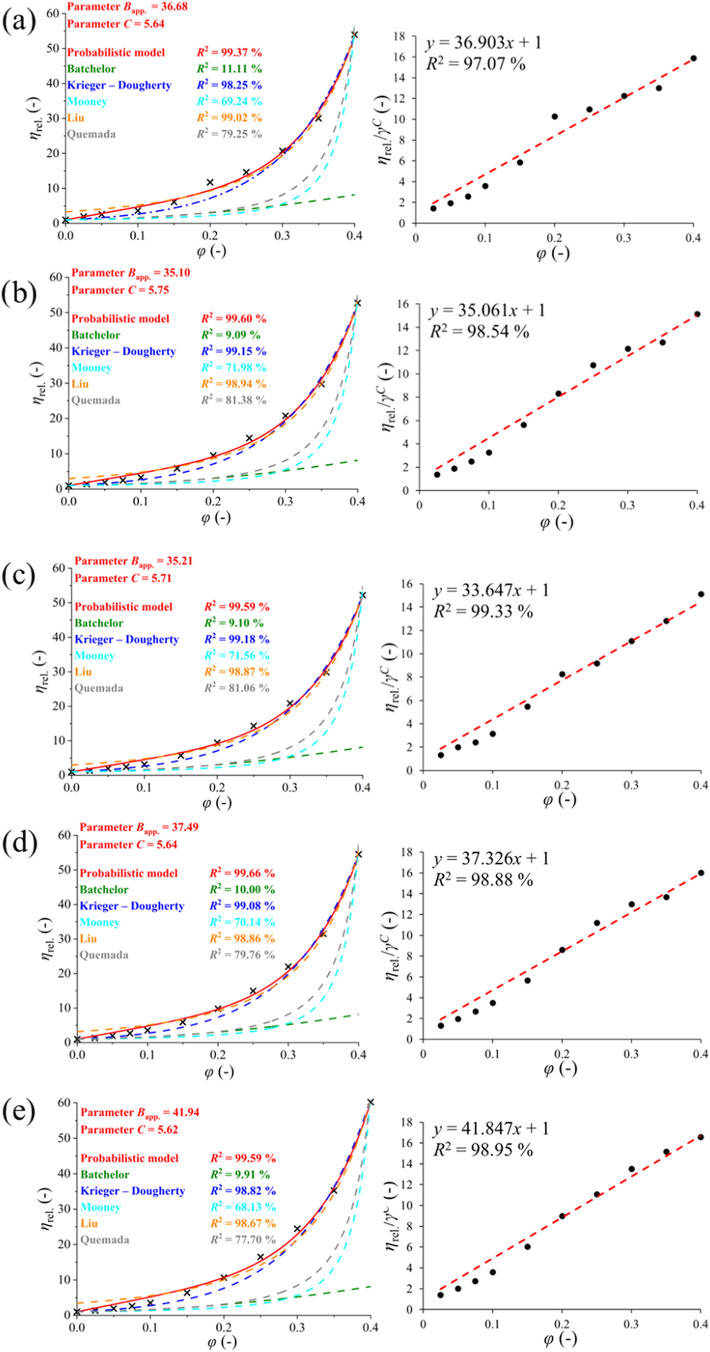
Graph of the dependence of relative viscosity on volume filling (left) with evaluation of physical consistency of the fit (right) for silica sand–CMC samples for (a) 25 °C; (b) 30 °C; (c) 35 °C; (d) 40 °C; (e) 50 °C measured at shear rate 10 s^–1^ (data obtained from the work of Sokola et al.,^51^ no graphics were reused).

Three models originating from Einsteińs limit (Batchelor, Krieger-Dougherty, and Mooney) were compared with the proposed model. As shown in Figures 3 and 4, the proposed model exhibited the best correlation with the data and achieved the highest *R* ^2^. The Batchelor’s model, due to its simple prescription, showed a very low correlation with the data, indicating its suitability for lower-volume fillings.^52^ Because there is no empirical fitting constant in the formulation, the model’s predictions come directly from the physics embedded in the theory, rather than being forced by a fit parameter.^17^ Also, the Mooney model frequently overpredicts relative viscosity values compared to experimental data across many systems,^37^ due to its single-exponential structure^53^ and tendency to overpredict values at high loadings,^26^ as shown in Figures 3 and 4. Consistent with our results, Horri et al.^15^ also reported a strong positive deviation of relative viscosity at higher volume filling. Finally, the Krieger–Dougherty model shows the highest correlation, specifically 94.77–96.60% for the 8Y-ZrO_2_–PEG system and 98.25–99.18% for the silica sand – CMC system. As shown in Figures 3 and 4, the Krieger–Dougherty model systematically tends to underpredict relative viscosity values in the low-to-medium range.^37,52,54^ Also, adding a dispersant to the system 8Y-ZrO_2_ – PEG influences the quality according to the Krieger–Dougherty model, developed for hard, rigid spheres,^55^ and adsorbed dispersant layers affect the effective volume.^56^

Two commonly used empirical models in ceramics technology, namely the Quemada model with prescription^57^

14 $$\eta_{rel.}={\left(1-\frac{1}{2}k\varphi \right)}^{-2}$$

and Liu model^58^ with the formula

15 $$\eta_{\mathrm{rel}.}={\left[a{(\varphi }_{\max}-\varphi )\right]}^{-n}$$

are used to describe rheological data. Constants *k*, *a*, and *n* are purely empirical and do not have an explicit physical interpretation.^58–60^ As stated by Pal,^26^ the Quemada model generally underpredicts viscosity at higher concentrations. The squared Pearson correlation coefficient of Liús model is reduced due to deviations predominantly in the domain of lower-volume fillings. Such deviations yield elevated relative viscosity values. Furthermore, discrepancies in the application of the Liu model have been documented in previous research.^15,61^

To strengthen the statistical testing of the proposed model, the Akaike Information Criterion (AIC) and the Bayesian Information Criterion (BIC) were used to compare the E^2^RG model with 4 tested models (besides the Batchelor polynomial fit). The AIC is defined as^62^

16 $$\mathrm{AIC}=n\cdot \ln\frac{\mathrm{RSS}}{n}+2k$$

where *n* represents the number of observations, *k* is the number of parameters in the model, and *RSS* represents the residual sum of squares.

Bayesian Information Criterion is defined as^63^

17 $$\mathrm{BIC}=n\cdot \ln\frac{\mathrm{RSS}}{n}+k\cdot \ln\left(n\right)$$

Lower AIC values indicate a better trade-off between accuracy and complexity, particularly when the objective is predictive accuracy. On the other hand, BIC imposes a stronger penalty for model complexity than AIC.^64^ By subtracting the AIC and BIC values of the proposed model and the compared model, predictive accuracy can be determined (providing a measure of the strength of evidence for each model relative to the best model).^65^ As shown in Tables 1 and 2, the proposed E^2^RG model yielded negative values (indicating better suitability) in all tested cases, supporting statistical testing and demonstrating its suitability for fitting rheological curves.

**Table 1:** △AIC and △BIC Values for 8Y-ZrO_2_–PEG Suspensions

model	△AIC (25 °C)	△BIC (25 °C)	△AIC (30 °C)	△BIC (30 °C)	△AIC (35 °C)	△BIC (35 °C)	△AIC (40 °C)	△BIC (40 °C)	△AIC (50 °C)	△BIC (50 °C)
Krieger- Dougherty	–11.8	–11.8	–13.1	–13.1	–14.5	–14.5	–11.3	–11.8	–14.5	–14.5
Mooney	–49.0	–48.5	–35.5	–37.0	–36.3	–37.8	–40.2	–37.7	–35.6	–37.1
Liu	–13.5	–13.5	–35.5	–37.0	–13.3	–13.3	–9.2	–9.6	–12.7	–12.7
Quemada	–29.7	–31.2	–31.6	–33.1	–32.5	–34.0	–36.4	–37.9	–13.1	–10.2

**Table 2:** △AIC and △BIC Values for Silica Sand −CMC Suspensions

model	△AIC (25 °C)	△BIC (25 °C)	△AIC (30 °C)	△BIC (30 °C)	△AIC (35 °C)	△BIC (35 °C)	△AIC (40 °C)	△BIC (40 °C)	△AIC (50 °C)	△BIC (50 °C)
Krieger-Dougherty	–54.1	–52.1	–66.6	–66.6	–7.8	–7.8	–38.6	–60.6	–66.4	–66.4
Mooney	–36.2	–37.3	–42.7	–44.3	–42.9	–44.4	–44.8	–46.3	–44.0	–45.5
Liu	–6.0	–5.1	–10.6	–10.6	–11.3	–11.3	–12.8	–12.8	–13.0	–13.0
Quemada	–32.3	–33.4	–38.2	–39.8	–38.4	–40.0	–40.5	–42.1	–40.1	–41.6

For further model validation, two photocurable zirconia systems from research^66^ with shear rates of 100 s^–1^ and 200 s^–1^ were fitted by the proposed model, providing high correlation (see Figure 5). Evaluation of shear rates is important in ceramics technology, especially for ceramic suspensions used in advanced manufacturing processes such as Direct Ink Writing (DIW), tape casting, or extrusion-based 3D printing, which are subjected to high shear rates during processing - typically over 100 s^–1^.^67,68^

**Figure 5:**
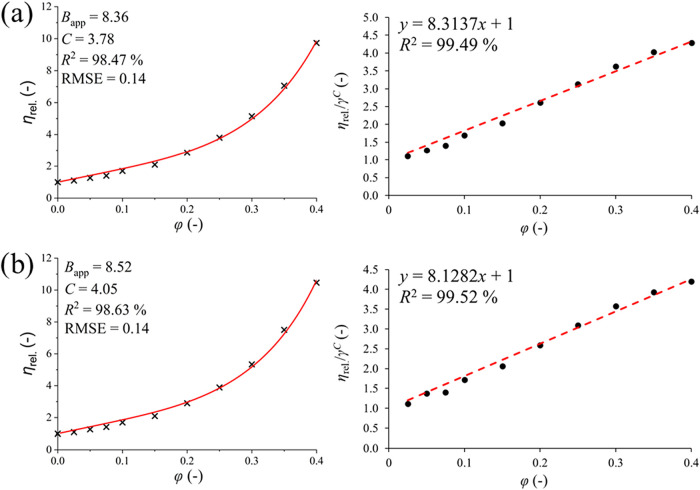
Graph of the dependence of relative viscosity on volume filling (left) with evaluation of physical consistency of the fit (right) for 3Y-ZrO_2_–acrylic resin samples measured at shear rate (a) 100 s^–1^; (b) 200 s^–1^ (data obtained from the work of Sokola et al.,^66^ no graphics were reused).

One of the key advantages of the E^2^RG model is its intrinsic verifiability, rooted in the Gaussian Allometric Evolution Law (GAEL) principle. After applying the correction term, linearization enables verification of whether the fit has correctly separated these components: a high *R* ^2^ across different temperatures and shear rates confirms not only numerical agreement but also the physical consistency of this partition. This feature distinguishes E^2^RG from conventional empirical models by providing an intrinsic mechanism for validating the physical meaning of the fitted parameters. Physical consistency (see Figures 3, 4, and 5, right columns) was achieved, with values exceeding 97%, confirming that the proposed model can successfully fit the data and that the two presented constants are physically consistent.

While the quality of the nonlinear fit (eq 6) demonstrates the mathematical correctness of E^2^RG, physical interpretability requires confirming that the fitted parameters retain their intended meaning rather than acting as arbitrary constants. This verification is enabled by the linearized form (eq 13), which cleanly separates the Einstein-form (eq 7) contribution from the interaction-driven term. When the resulting linear relation maintains a high correlation, the fit is internally consistent, and the parameters *B*_app_. and *C* reliably reflect distinct physical aspects of the system. Finally, the ratio *B*_app._ serves as an indicator of system individuality, expressing how strongly particle interactions amplify the effective contribution compared to the ideal Einstein value. In this way, the validation step becomes more than a numerical fit: it becomes a check of physical consistency, confirming that the fitted parameters retain their intended meaning within the theoretical framework. This step should therefore be understood as a consistency check within the model, rather than an independent validation of its predictive capability.

Any perturbation of the system (in concentration, temperature, or shear rate) temporarily displaces the distribution of interaction constraints from its Gaussian CLT-consistent form. Because γ­(φ) arises as the Gaussian limit of many weak multiplicative interactions, the system naturally relaxes back toward this form along the statistically shortest path, restoring its characteristic CLT - consistent statistical form. The fact that this same functional form (eq 3) consistently satisfies all physical and mathematical constraints, from the dilute Einstein limit to the concentrated-regime asymptotics, supports the view that the formulation reflects an inherent property of the system rather than an imposed empirical choice. Furthermore, the Gaussian term reflects a maximum-probability perspective: the Statistical Flow Limit corresponds to the concentration at which the probabilities of successful and failed momentum-transfer events become equal, marking a statistically neutral state of flow.

The model is not restricted to spherical particles, as it captures the macroscopic response through cumulative interaction constraints rather than explicit particle geometry. Particle anisotropy is expected to influence primarily the parameter *C*, reflecting an increased density and correlation of interaction constraints, while preserving the overall monotonic character of the concentration dependence. From a practical perspective, the E^2^RG formulation can be used to describe and compare concentration-dependent rheological behavior across different suspension systems. By fitting experimental data, the model could provide system-specific parameters (*B*_app._ and *C*) that characterize the influence of particle interactions and enable comparison between different formulations or processing conditions.

## Conclusion

The E^2^RG model provides a compact and physically grounded framework for describing the transition from ideal to interaction-constrained flow in concentrated suspensions. By preserving the Einstein limit while introducing a Gaussian weighting factor, the formulation captures the cumulative effect of particle-interaction constraints without relying on empirical divergences or arbitrary concentration-based exponents. This factor reflects the CLT-driven convergence of multiplicative microconstraints toward a Gaussian form, rather than an empirical assumption. The two model parameters, *B*_app._ and *C*, represent complementary projections of interaction-driven behavior within the formulation and, being obtained by fitting, should be interpreted as system-specific quantities rather than independently measurable physical constants.

The Statistical Flow Limit at φ_max_ ≈ 0.62 offers a physically meaningful boundary at which continuous macroscopic flow becomes statistically unsustainable, while microscopic mobility and dissipation remain possible. Unlike classical singular models, which impose a formal divergence at φ_max._, E^2^RG provides a consistent description of flow cessation as a statistical transition rather than material solidification. By embedding interaction-driven constraints in a mathematically transparent form, the E^2^RG model offers improved descriptive capability, intrinsic verifiability through linearization, and a conceptually coherent alternative to traditional extensions of Einstein’s equation.

## Data Availability

The data used in this study are available at: DOI:10.5281/zenodo.18374890

## Notes on Notation

**All Parameters Listed Below are Dimensionless Unless Stated Otherwise. The Following List Summarizes the Notation Employed in the Present Work:**
φ: Particle volume fraction
φ_max._: Statistical Flow Limit (SFL), probability-neutral jamming threshold
η_rel._: Relative viscosity at a shear rate of 50 s^–1^
η_rel._^*EF*^: Einstein Form of the relative viscosity
*B*: Ideal intrinsic viscosity (2.5 for hard spheres)
*B*_app._: Apparent intrinsic viscosity; a system-specific effective Einstein coefficient reflecting the collective (correlated) contribution of interacting particle groups, rather than the ideal single-particle value B.
γ­(φ): Gaussian weighting factor, eq (3), emerging from CLT; a smooth, monotonic Sigmoid-type function describing the cumulative effect of multiplicative interaction constraints
*C*: Scaling exponent of Gaussian deformation (interaction intensity); it reflects the system-specific degree of correlated particle interactions that reduce the likelihood of successful momentum transfer.
γ­(φ)*^C^*: Allometric (nonlinear) amplification of the Gaussian weighting factor; the scaling exponent *C* magnifies the statistical influence of γ­(φ), transforming its monotonic rise into the accelerated growth characteristic of concentrated suspensions.
